# Cerebrum, liver, and muscle regulatory networks uncover maternal nutrition effects in developmental programming of beef cattle during early pregnancy

**DOI:** 10.1038/s41598-021-82156-w

**Published:** 2021-02-02

**Authors:** Wellison J. S. Diniz, Matthew S. Crouse, Robert A. Cushman, Kyle J. McLean, Joel S. Caton, Carl R. Dahlen, Lawrence P. Reynolds, Alison K. Ward

**Affiliations:** 1 Department of Animal Sciences, Center for Nutrition and Pregnancy, North Dakota State University, Fargo, ND USA; 2 USDA, ARS, U.S. Meat Animal Research Center, Clay Center, NE USA; 3 Department of Animal Science, University of Tennessee, Knoxville, TN USA

**Keywords:** Gene expression profiling, Transcriptomics

## Abstract

The molecular basis underlying fetal programming in response to maternal nutrition remains unclear. Herein, we investigated the regulatory relationships between genes in fetal cerebrum, liver, and muscle tissues to shed light on the putative mechanisms that underlie the effects of early maternal nutrient restriction on bovine developmental programming. To this end, cerebrum, liver, and muscle gene expression were measured with RNA-Seq in 14 fetuses collected on day 50 of gestation from dams fed a diet initiated at breeding to either achieve 60% (RES, n = 7) or 100% (CON, n = 7) of energy requirements. To build a tissue-to-tissue gene network, we prioritized tissue-specific genes, transcription factors, and differentially expressed genes. Furthermore, we built condition-specific networks to identify differentially co-expressed or connected genes. Nutrient restriction led to differential tissue regulation between the treatments. Myogenic factors differentially regulated by *ZBTB33* and *ZNF131* may negatively affect myogenesis. Additionally, nutrient-sensing pathways, such as mTOR and PI3K/Akt, were affected by gene expression changes in response to nutrient restriction. By unveiling the network properties, we identified major regulators driving gene expression. However, further research is still needed to determine the impact of early maternal nutrition and strategic supplementation on pre- and post-natal performance.

## Introduction

The relevance of optimal maternal nutrient supply in the periconception period and during pregnancy has received close attention due to growing evidence showing its impact on offspring development^1–3^. Imbalance in the availability of uterine-derived histotroph^4^ as well as placental nutrient supply, either by overfeeding or underfeeding, leads the fetus to physiological and metabolic changes with long-term consequences^5–7^. Nutritional insults affect energy partitioning among metabolically active target tissues, such as the liver and muscle^8^, affecting economic traits such as lactation, reproduction, meat quality, body composition, and carcass weight^5,9,10^. These adverse long-term consequences could decrease the livestock industry’s profitability as the negative performance may be epigenetically inherited across generations^11^.

The brain, liver, and muscle tissues have different priorities in nutrient partitioning when limited resources are available, affecting their development and functionality^8,12^. The liver plays a key role in body energy metabolism^13^, whereas the brain, by regulating liver metabolism, prioritizes its own glucose needs over those of other tissues^14^. Thus, with restricted energy availability, more glucose is allocated for the functioning of the central nervous system^14^, potentially impairing muscle development and reducing muscle mass^12^. Reduced number of myofibers and liver sizes have been reported in nutrient restricted sheep fetuses and adults, respectively^15,16^. Furthermore, intrauterine growth restricted bovine fetuses showed increased brain weight as a percentage of fetal body weight in response to early maternal undernutrition^6^. Altogether, the nutritional regulation of fetal programming underlies a complex genomic regulatory network that modulates gene expression and leads to changes in tissue structure and function^2,3,8^. Differences in gene expression patterns and regulation of epigenetic mechanisms were reported as underlying the tissue-specific metabolic changes observed in nutrient restricted offspring^1,15–17^.

Previously, our group showed that the exposure of pregnant cows to a nutritional insult in early pregnancy changed fetal liver, cerebrum, and muscle gene expression profiles, suggesting a possible compensatory growth in response to reduced nutrient availability^17^. Likewise, candidate genes involved with myogenesis, muscle differentiation, and hepatic metabolism were reported as differentially expressed as a consequence of maternal nutrition^1,15,18^. Despite the knowledge provided by differential expression analysis, this approach does not consider the multiple interactions involved with gene expression regulation^19^ that are central to phenotype determination. To overcome this limitation, gene networks can be used successfully as a complementary framework to untangle the mechanisms and unravel the effects of maternal nutrition on fetal programming^8^. By measuring gene co-expression similarity, we can capture the relationship among genes across experimental conditions or tissue types and decipher the biologically-related gene functions^19–21^. Furthermore, we can assess the network ‘rewiring’ and the differences in gene–gene interactions driving changes in gene expression in response to external stimuli^20,22^.

Although we have a growing understanding of the molecular and physiological basis of fetal programming, it is still unclear to the extent these differences can be attributed to the coordinated function of genes and the regulation of specific pathways. Therefore, we hypothesized that maternal nutrient restriction from breeding to day 50 of gestation would negatively affect fetal cerebrum, liver, and muscle development and function through differential gene regulation. Herein, by measuring the gene–gene interactions, we uncovered the regulatory interplay between genes and showed that these fetal tissues were differentially regulated in response to maternal nutrition.

## Results

**Figure 1:**
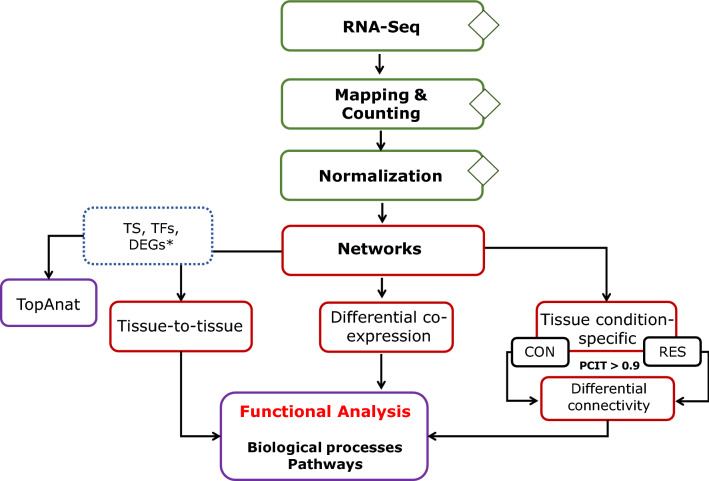
Bioinformatics workflow of the multi-tissue RNA-Seq-based gene co-expression network. TS—tissue-specific genes; *DEGs—differentially expressed genes^17^; TFs—transcription factors; CON—control; RES—restricted.

We applied tissue-to-tissue and tissue condition-specific co-expression network approaches to infer the regulatory relationships between genes and to shed light on the putative mechanisms underlying the effects of early maternal nutrient restriction on bovine developmental programming (Fig. 1). Previously, Crouse et al.^17^ reported 373 differentially expressed genes (DEG) from fetal liver (*n* = 201), muscle (*n* = 144), and cerebrum (*n* = 28) by contrasting control (CON, *n* = 7) and nutrient restricted (RES, *n* = 7) groups. Herein, we report significant differentially connected and differentially co-expressed genes in 50-day fetuses. Likewise, we identified putative regulators driving the differential gene expression between the groups and the over-represented biological pathways.

### RNA sequencing data analysis

After quality control, on average, 92.8%, 89.9%, and 93.0% of unique reads from the cerebrum, liver, and muscle tissues, respectively, were mapped to the reference genome. A summary of sequencing throughput and mapping rates per sample and tissue is reported in Tables S1, S2, and S3. Mapped reads saturation plot showed that, on average, 92% of the genes were identified, supporting an adequate read-depth per tissue in the current study (Figure S1). The multi-tissue normalization approach (*see **Methods*) identified 17,164 genes out of 27,607 reported on the *Ensembl* annotation file that were clustered by tissue, as shown in Figure S2.

### Tissue-specific genes

**Figure 2:**
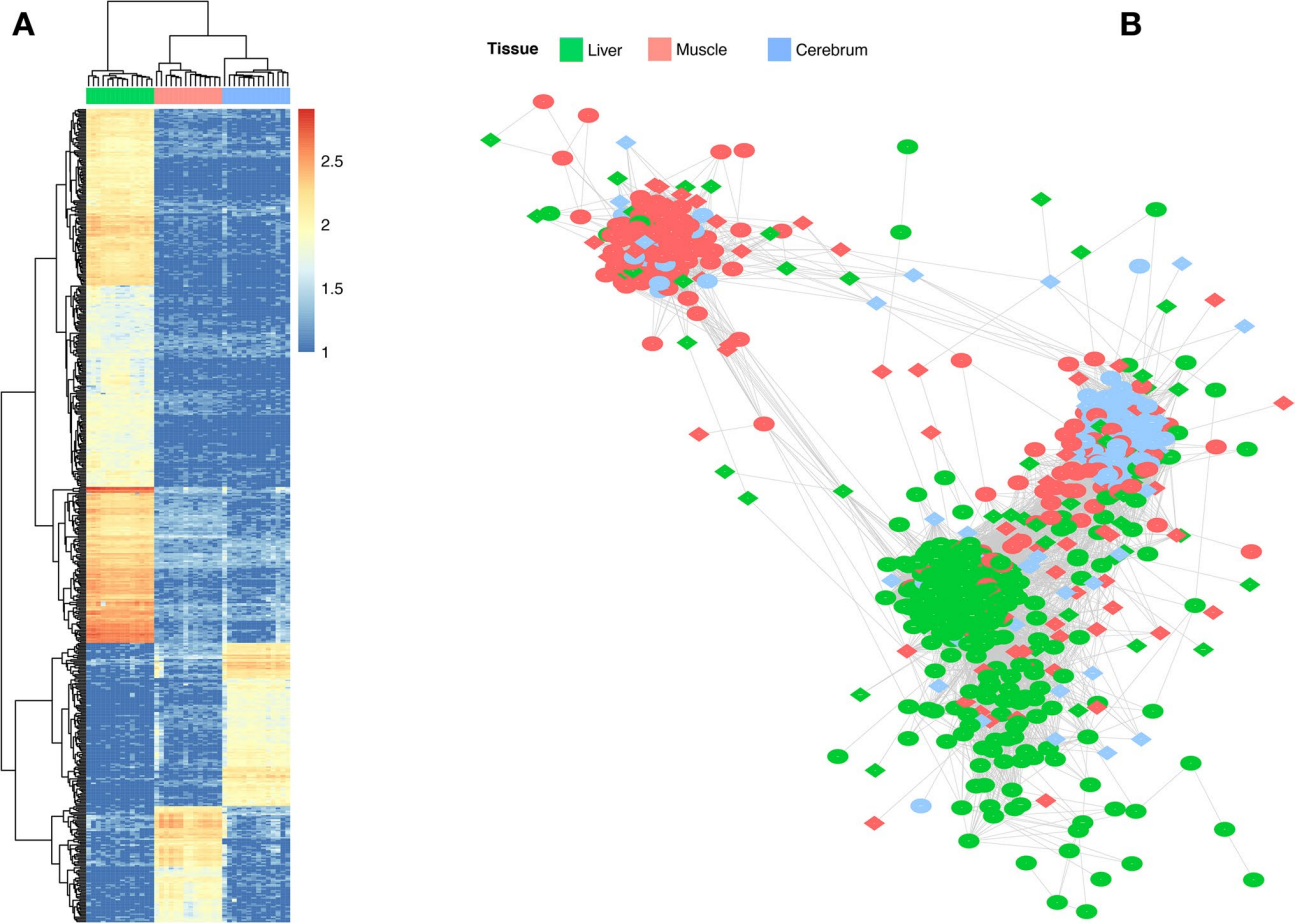
Tissue-to-tissue co-expression network of the bovine fetal organ transcriptome. (**A**) Heat map of 553 tissue-specific genes from the liver, muscle, and cerebrum tissues. (**B**) Co-expression network of 992 significantly co-expressed genes. Only nodes with a correlation greater than |0.9| are shown. Overlapped genes between analysis were colored based on the tissue with its maximum expression. Transcription factors are represented by a diamond shape. Nodes with few connections not linked to the main network are not showed. Heat map was constructed using pheatmap v.1.010.^93^ on R^79^, whereas gene network was created on Cytoscape v.3.7^89^.

Based on the Tau index^23^, we identified 553 tissue-specific (TS) genes (τ ≥ 0.8). The liver (*n* = 363) and cerebrum (*n* = 111) expressed the greatest number of TS genes, followed by muscle (*n* = 79) (Fig. 2A). The *NEUROD6*, *PFKFB*, and *MYOG* genes were amongst the TS genes with the greatest τ values for the cerebrum, liver, and muscle, respectively. Furthermore, 19 and 15 TS genes were reported as differentially expressed for the liver and muscle. To support our approach, we carried out a tissue over-representation analysis. As expected, the genes were indeed over-represented for the tissues where they are preferentially expressed [prefrontal cortex, adjusted *p-value* (*adj.pvalue*) = 4.59 E−10; liver, *adj.pvalue* = 4.58E−05; and skeletal muscle, *adj.pvalue* = 2.11 E−05]. Additionally, gene ontology (GO) terms highlighted biological processes such as forebrain development (*adj.pvalue* = 2.26E−21), hemostasis (*adj.pvalue* = 1.68E−16), and muscle organ development (*adj.pvalue* = 1.55E−21). The over-represented Uberon ontology terms, the directed acyclic graphic, and the GO terms are reported in Tables S1, S2, and S3.

### Key regulatory genes (transcription factors, TFs)

Regulatory impact factors, RIF1 and RIF2^24^, were used to identify TFs potentially modulating the expression of DEG and TS genes. Based on those metrics, we identified 129, 119, and 90 TFs for liver, muscle, and cerebrum tissues, respectively, grouped into 52 families (*p* ≤ 0.01). Shared TFs were identified between cerebrum and muscle (*n* = 10), muscle and liver (*n* = 8), and liver and cerebrum (*n* = 7). The *HOXA4* (*z-score* =  − 6.88), *ZBTB33* (*z-score* =  − 4.37), and *GATA1* (*z-score* =  − 3.85) genes showed the most extreme value for RIF1 in liver, muscle, and cerebrum, respectively. A list of all RIF1 and RIF2 significant TFs is presented in Tables S1, S2, and S3. Herein, those genes encoding TFs and identified as key regulators will be called TFs throughout the text. The over-represented BP and pathways in which the TFs are involved with DEGs and TS genes are reported in the Tab8 of the Supplementary Tables S1 (muscle), S2 (cerebrum), and S3 (liver).

### Tissue-to-tissue co-expression network

To build a network across tissues based on the PCIT framework, we prioritized 1,160 unique informative genes considering the following criteria: (1) DEG between RES and CON groups; (2) tissue-specific; and (3) key TFs based on RIF1 or RIF2. Based on that, a significant network with 992 nodes and 105,449 interactions (r >|0.9|) was retrieved (Table S4, Fig. S3). The network had a high clustering coefficient (0.8) and was clustered by tissue (Fig. 2B), mainly driven by TS genes, followed by DEGs and TFs. Genes from the liver showed higher average connectivity (284.5) compared to the cerebrum (144.7) or muscle (100.06). The *GATA1*, *HSF2*, and *PLEK* TFs showed the greatest connectivity within liver, muscle, and cerebrum tissues, respectively. Furthermore, these genes shared all the first neighbors of one another, creating a sub-network with 427 nodes and 81,308 connections (Fig. S4).

### Differential gene co-expression network analysis

**Figure 3:**
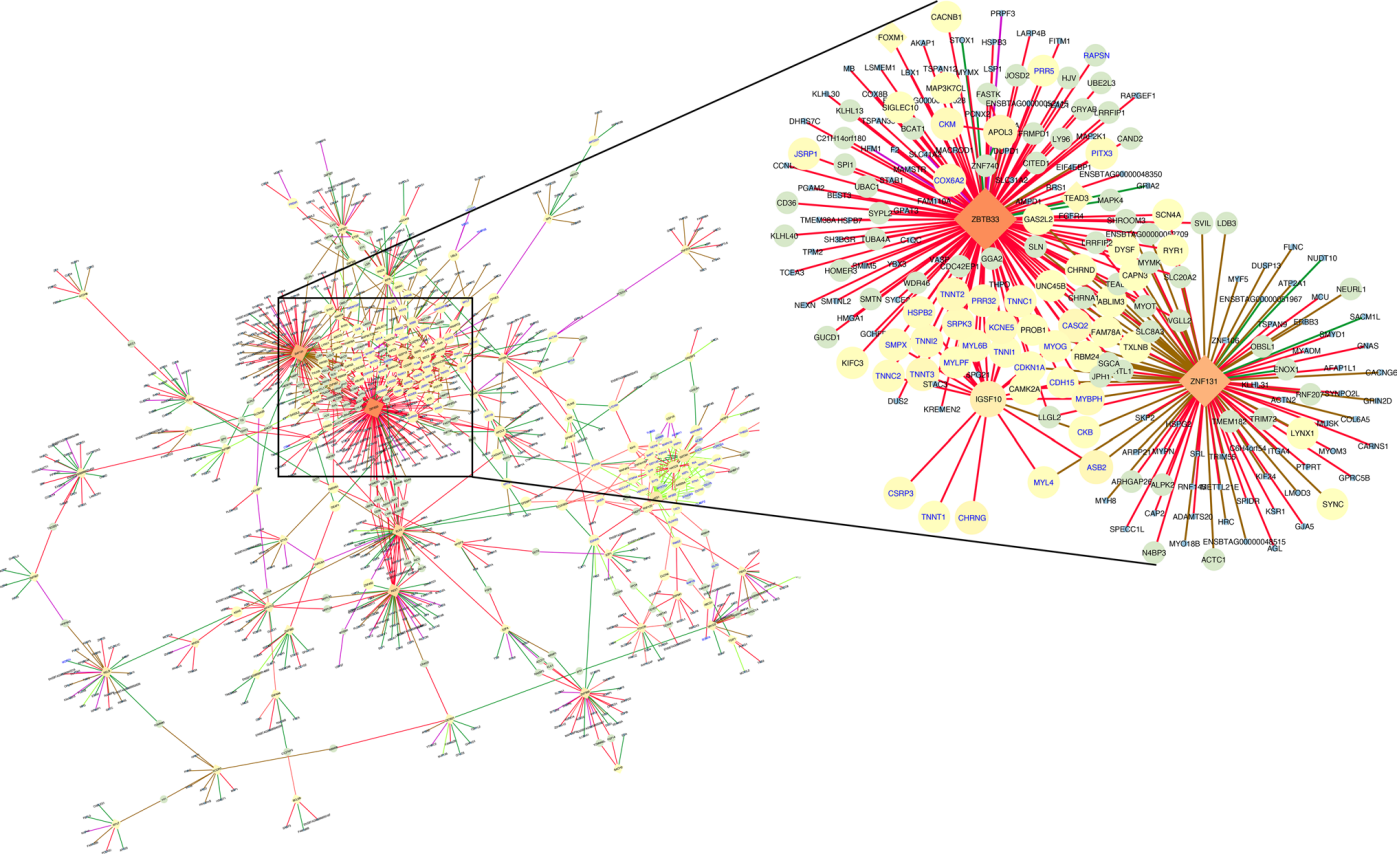
Muscle regulatory network of differentially co-expressed genes from the bovine transcriptome. Nodes are genes with significant changes in the correlation between control and nutrient restricted fetus (*q* ≤ 0.05). The node size and color (from light to dark) are proportional to the number of connections for each gene. Nodes with few connections not linked to the main network are not shown. Transcription factors are represented by a diamond shape. Differentially expressed genes are labeled in blue. Edges are colored based on the differential correlation class (+/− , red; +/0, salmon; − /+ , green; − /0, green-yellow; 0/+ , magenta; 0/− , brown). Gene network was created on Cytoscape v.3.7^89^.

To uncover the functional relationship between gene pairs across conditions, we applied a differential gene co-expression analysis for each tissue separately. From muscle, we identified 17,282 differentially co-expressed (DC) gene pairs (corresponding to 7,479 unique genes out of 11,439 tested; *q* ≤ 0.05; Table S1). Among them, 101 and 91 genes were identified as DEGs and TFs, respectively. Figure 3 shows the network of DC genes filtered by those gene pairs assigned either DEG or TF for muscle. We used the connectivity as a measure of centrality to select the genes topologically important in the network (“hubs”). Accordingly, 225 DC genes were identified as hubs (*p* < 0.05). The top five hub genes included *IGSF10*, *ZBTB33*, *CD2AP*, *PLEKHG1*, and *ZBTB1*, and were associated with more than 120 genes in muscle. The DEGs were mainly differentially co-expressed with the genes *ZBTB33* (*n* = 24), *IGSF10* (*n* = 24), *HACD3* (*n* = 16), and *ZNF131* (*n* = 8). Interestingly, the co-expression of *ZBTB33* and *ZNF131* with the DEGs were classified as inversely correlated between the maternal diet groups based on the correlation classes proposed by the R-package DGCA^22^ (Figs. 3, S5).

**Figure 4:**
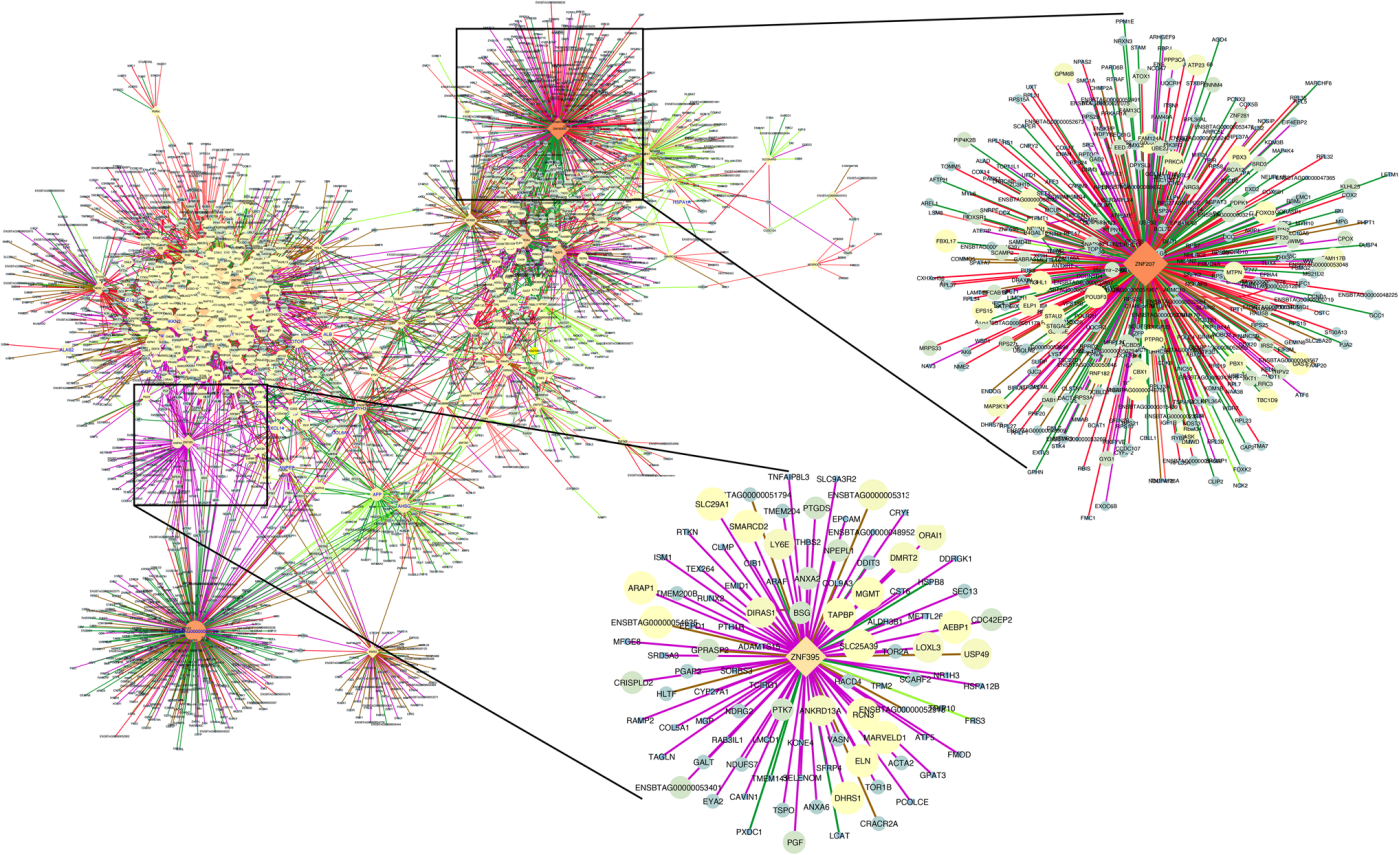
Cerebrum regulatory network of differentially co-expressed genes from the bovine transcriptome. Nodes are genes with significant changes in the correlation between control and nutrient restricted fetus (*q* ≤ 0.05). The node size and color (from light to dark) are proportional to the number of connections for each gene. Nodes with few connections not linked to the main network are not showed. Only those gene pairs assigned as DEG, TS, or TF are shown. Transcription factors are represented by a diamond shape. Differentially expressed genes are labeled in blue. Edges are colored based on the differential correlation class (+/− , red; + /0, salmon; − /+ , green; − /− , deep sky blue; − /0, green-yellow; 0/+ , magenta; 0/− , brown). Gene network was created on Cytoscape v.3.7^89^.

We found 284,738 DC gene pairs by applying the same approach to the cerebrum. To reduce the data dimensionality, these pairs were then filtered by DEG, TS, or TF genes. Based on that, 14,423 DC pairs (4676 unique genes) (*q* ≤ 0.05) remained for further analysis (Table S2). The TS genes *ELAV4*, *PAK5, SLC17A6, GPR12*, and *CLVS2,* were the most differentially co-expressed ones (hubs). Among the 61 genes classified as hubs (*p* < 0.05), 21 were TFs identified as putative regulators of brain gene expression. From these TFs, *ZNF207*, *ETS2*, and the novel gene *ENSBTAG00000003447* were highlighted as targeting 412, 250, and 248 genes (Fig. 4). Unlike the muscle, no clear pattern of correlation was observed for TFs and DEGs differentially co-expressed between conditions.

**Figure 5:**
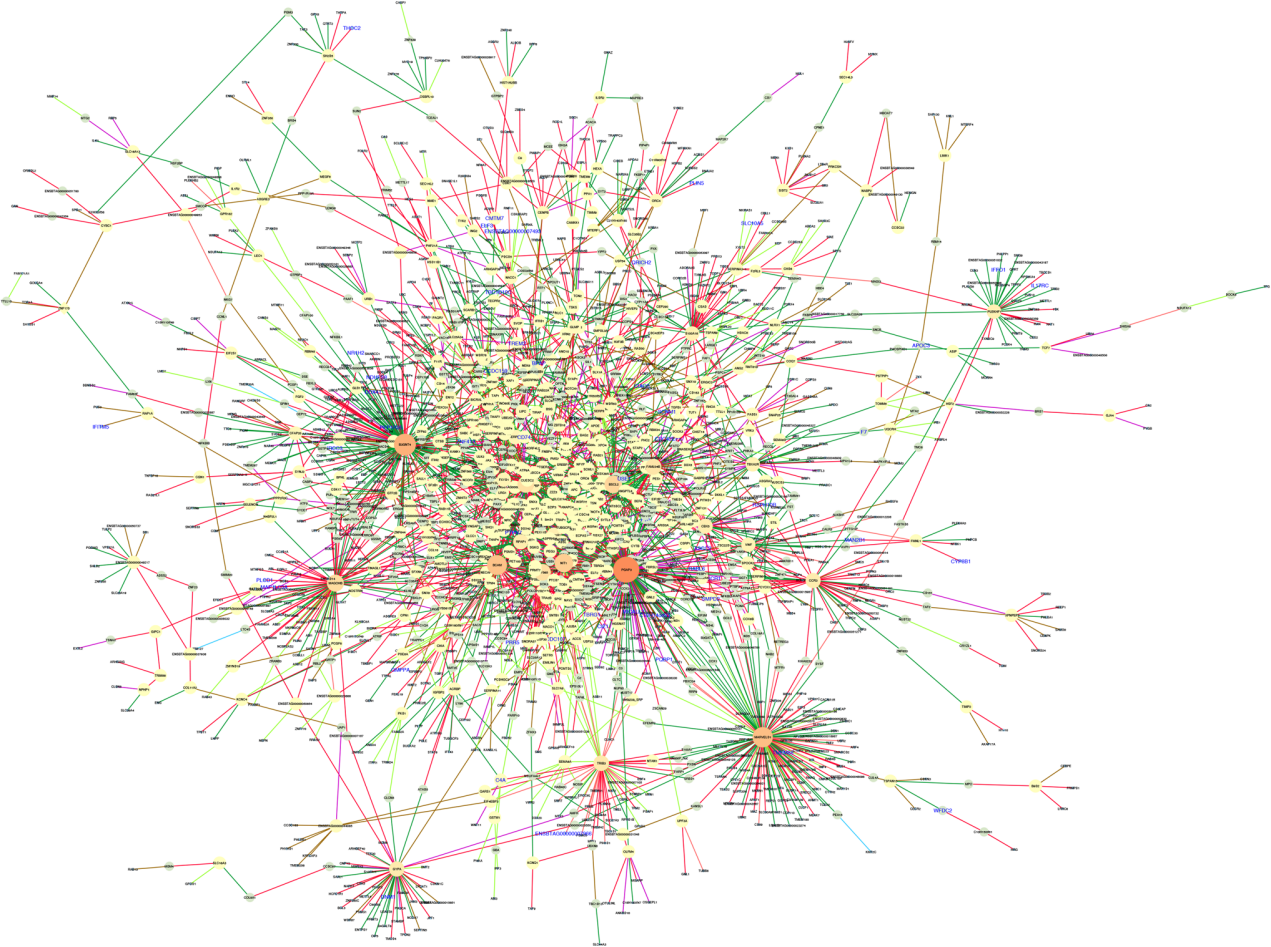
Liver regulatory network of differentially co-expressed genes from the bovine transcriptome. Nodes are genes with significant changes in the correlation between control and nutrient restricted fetus (*q* ≤ 0.05). The node size and color (from light to dark) are proportional to the number of connections for each gene. Nodes with few connections not linked to the main network are not showed. Transcription factors are represented by a diamond shape. Differentially expressed genes are labeled in blue. Edges are colored based on the differential correlation class (+/− , red; + /0, salmon; − /+ , green; − /0, green-yellow; 0/+ , magenta; 0/− , brown). Gene network was created on Cytoscape v.3.7^89^.

Regarding the liver, we identified 2,989 DC pairs (2319 unique genes) that included 56 DEGs and 23 TFs (*q* ≤ 0.05) (Table S3). The top five hub genes among the 27 identified included: *PGAP3, B3GNT4, MARVELD1, BCAM*, and *NIT1*. The *PGAP3* gene stood out as the main DC gene with 11 DEGs. These pairs were classified as positively correlated in the RES group and negative or not significant in the CON group (−/+ or 0/+) (Fig. 5). Additionally, the *ZNF175, HSF1, and HSF2* TFs were among the hubs.

### Differential connectivity analysis

**Figure 6:**
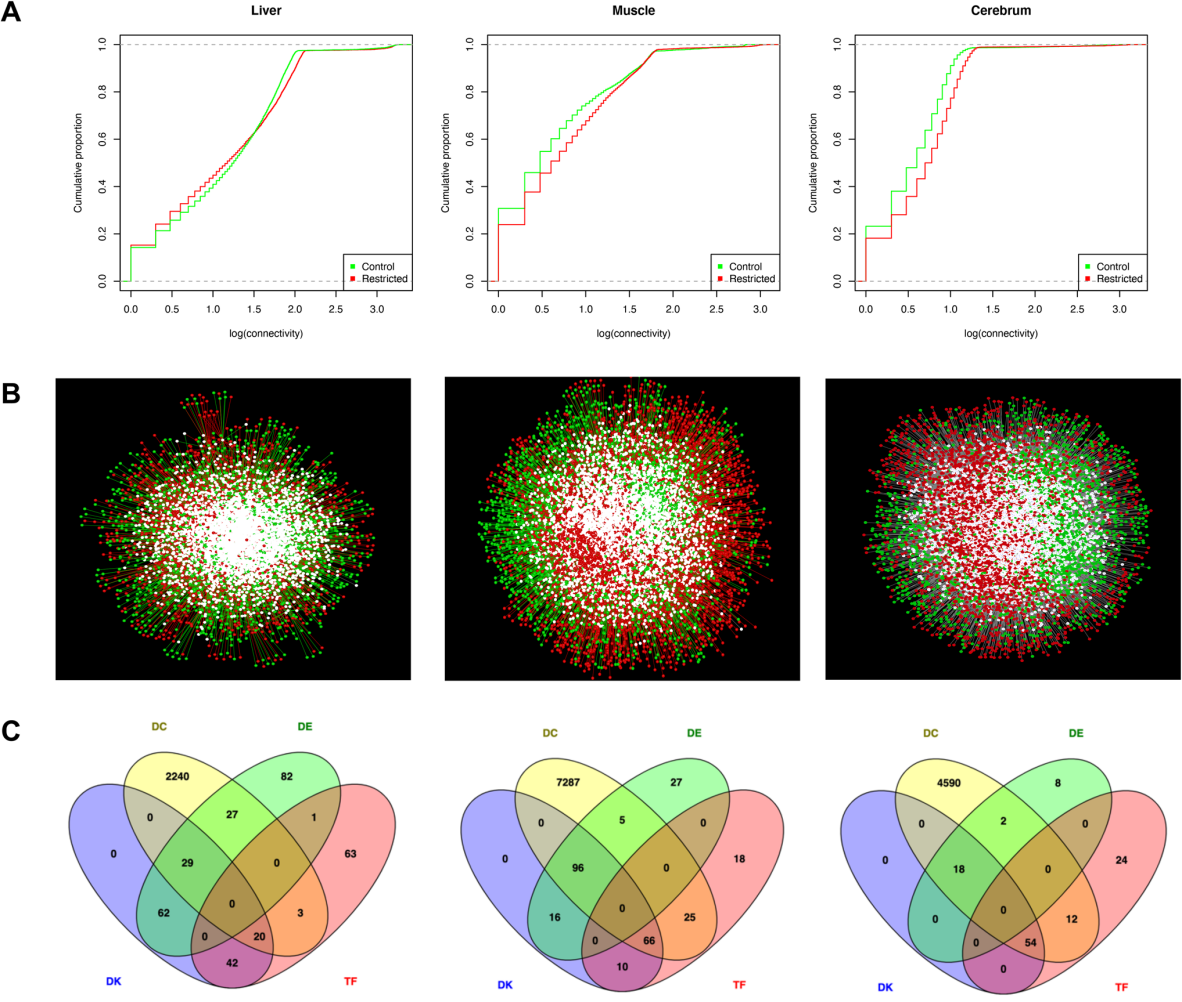
Network topology of co-expressed genes between control and nutrient restricted fetuses from the liver, muscle, and cerebrum tissues in bovine. (**A**) Cumulative distribution functions of the gene connectivity. (**B**) Central reference union networks. Only those gene pairs assigned as DEG or TF are shown. Unique nodes are shown in green (control) or red (restricted). Shared nodes are shown in white; (**C**) Overlapping genes among the different analyses. TF—transcription factors; DE – differentially expressed gene; DC—differentially co-expressed; and DK—differentially connected. Cumulative distribution was created on R v.3.5.1^79^; Gene network was created on Cytoscape v.3.7^89^; and Venn diagram was created using Venny v.2.1.^94^ (https://rb.gy/jxxufy).

To identify condition-specific connections, we inferred networks in the CON and RES groups separately for each tissue. Based on the concept of differential connectivity^25^, we identified the most rewired genes between the groups (Tables S1, S2, S3). Despite the similar topological behavior, overall, we found a gain of connectivity in networks from RES (Fig. 6A,B). These networks followed a scale-free model with R-squared ranging from 0.67 to 0.89.

From the liver, the networks filtered for either DEG or TF genes resulted in 8,350 and 8,632 nodes and 261,099 and 231,270 edges for RES and CON groups, respectively. By contrasting the connectivity between the groups, we found 153 differentially connected (DK) genes (*p* < 0.05). Similarly, we identified 188 DK genes in muscle by contrasting 6,925 nodes and 63,105 edges with 8515 nodes and 94,186 edges from CON and RES groups, respectively. Regarding the cerebrum, the CON network was composed of 6,390 nodes and 29,531 edges, whereas the RES one had 7,555 nodes and 48,662 edges. Accordingly, we identified 72 DK genes (*p* < 0.05). Several DEGs from the liver and muscle tissues were also identified as DK (Fig. 6C).

### Functional over-representation analysis

Functional analysis revealed a total of 374, 212, and 143 significant biological process (BP) terms over-represented in the liver, muscle, and cerebrum gene lists, respectively (*adj.pvalue* ≤ 0.05). In the liver, common BP across DE, TFs, and TS genes included categories such as blood coagulation, immune response (defense and inflammatory response), and energy metabolism (fatty acid metabolic process and cellular glucose homeostasis). Tissue specific genes highlighted BP related to metabolism, transport, and blood coagulation, whereas TFs underlined regulation of RNA biosynthetic process and hormone metabolic process (Table S3). The pathway analysis included complement and coagulation cascade (*adj.pvalue* = 2.9E−13), metabolic pathways (*adj.pvalue* = 2.64E−02), and maturity onset diabetes of the young (*adj.pvalue* = 2.64E−02) (Table S3). Moreover, MAPK (*adj.pvalue* = 3.32E−02), non-alcoholic fatty liver disease (NAFLD) (*adj.pvalue* = 3.68E−02), and oxidative phosphorylation (*adj.pvalue* = 3.32E−02) pathways were retrieved from DC genes.

Regarding the muscle, the identified GO terms pointed out the muscle structure development and myotube and myoblast differentiation as common BP among the gene lists. Similarly, TS and TF genes included skeletal system morphogenesis and regulation of transcription from RNA polymerase II promoter (Table S1). Furthermore, FoxO (*adj.pvalue* = 8.32E−06), MAPK (*adj.pvalue* = 3.69E−09), and PI3K/Akt (*adj.pvalue* = 3.94E−07) signaling pathways were among the over-represented pathways from the DC genes.

From the cerebrum, central nervous system development (*adj.pvalue* = 1.68E−13) and brain development (*adj.pvalue* = 4.60E−08) were among the over-represented BP underlying the TS genes. The TF gene list retrieved BP such as cellular response to steroid hormone stimulus (*adj.pvalue* = 6.47E−07) and negative regulation of DNA binding transcription factor activity (*adj.pvalue* = 6.47E−07) (Table S2). Moreover, signaling pathways from the DC genes highlighted, for example, ribosome (*adj.pvalue* = 8.86E−21), PI3K-Akt (*adj.pvalue* = 3.19E−06), insulin (*adj.pvalue* = 3.11E−03), mTOR (*adj.pvalue* = 2.41E−04), and HIF-1 signaling pathways (*adj.pvalue* = 2.17E−03). Tissue-specific and overlapping pathways across tissues are showed in Figure S6.

## Discussion

Dietary restriction has a negative effect on the offspring’s development, metabolism and genome function with long-term consequences^2,3,5,9^. Previously, we reported a significant impact on genome-wide expression profiles underpinning tissue metabolism, accretion, and function in fetal muscle, liver, and cerebrum from dietary intake-restricted heifers^17^. Herein, based on the co-expression network, we uncovered the regulatory interplay between genes and shed light on the putative mechanisms underlying the effects of early maternal nutrient restriction on bovine developmental programming on day 50 of gestation. We found that these tissues were affected by dietary intake restriction leading to differences in transcriptional regulation between CON and RES groups. Furthermore, we pointed out TFs potentially modulating the expression of DEGs and TS genes.

Tissue specificity results from a tight regulation of gene expression^26^ driven by different regulatory mechanisms that include TFs and cross-tissue communication^20^. The TFs play a significant role in determining differential expression in response to nutrient availability^7^, although they are not themselves usually DEG^26^. We successfully mapped 338 TFs as putative regulators of TS and DEG. It is worth mentioning that these are not the only TFs regulating the tissue development and gene transcription; however, they were consistently most differentially co-expressed (RIF1) or could predict (RIF2) the abundance of the TS and DEG^24^. The regulatory role of these TFs on day 50 of gestation was perturbed due to maternal nutrient restriction, as evidenced by their identification either as DC or DK. The liver showed the greatest number of TS genes, followed by the cerebrum and muscle. Moreover, the greatest number of DEG and TF genes were found in the liver, emphasizing its wide range of essential metabolic functions.

To provide an overview of the gene expression relationship across the fetal tissues studied, we prioritized informative genes from TS, TF, and DEG analyses. This integrative framework allowed us to dissect the gene–gene interactions within and across tissues, uncovering new insights into how these relationships may be rewired due to maternal nutrition^8,27,28^. Our tissue-to-tissue network exhibited a small-world behavior^29^, mainly driven by the TS genes that showed a strong clustering. Based on this guilty-by-association assumption, we pinpointed tissue-specific functions related to tissue development, structure, and metabolism.

To have tissue genome-scale information, we identified specific co-expression differences between CON and RES groups based on DGCA and DK analyses. We identified hub genes with important biological roles. Furthermore, we found a gain of connectivity in the networks from fetuses from the RES dams. The rewiring of major regulators likely modulates the expression of gene targets as an adaptive response to nutrient availability, impacting tissue development. These findings are supported by the DGCA results that showed TFs differentially coordinating gene transcription between the groups. For example, we found transcriptional repressors in muscle, such as *ZBTB33*, *REST*, and *RCOR3*, more connected in the RES network. Likewise, we identified *ARID2,* whose protein is involved with chromatin remodeling that is important for metabolic programming^2^. Herein, we will mainly focus our discussion on the TFs considering their topological importance in the network for each tissue, the over-represented pathways, and their regulatory role in fetal tissue programming.

The liver plays a pivotal role in energy metabolism^13^. Energy imbalances affect liver function and trigger a cascade of events in insulin sensitive tissues, such as the brain and muscle^7,18^. As a response to a moderate maternal nutrient restriction, the fetuses from the RES group tended to have a reduced level of glucose in allantoic fluid^30^. In goats, Zhou et al.^18^ found several glucogenic amino acids (AA) differentially abundant in the liver of nutrient-restricted fetuses. Supporting these findings, we identified pathways related to glucogenic AA, such as arginine biosynthesis and tryptophan metabolism. Additionally, the liver is the main hematopoietic organ during pre-natal development^18^. We found *GATA1* as the most connected TF, and this gene is important for early hematopoiesis^31^. Furthermore, the liver is the primary site of complement component synthesis^32^. The complement system is essential for host defense, and its failure has been associated with adverse pregnancy and fetal growth restriction in humans^32^. The complement and coagulation cascade pathway, as well as several hematopoietic genes were pinpointed in our findings.

We identified master regulators of energy homeostasis, such as *FOXA3*, *PPARA*, and *SREBF2*. Among the pathways important for energy homeostasis, we found MAPK, which acts as a PPAR regulator^33^. The PPAR protein is suggested to have an adaptive role during tissue development in response to nutrient availability^7^. The transcriptional complex formed by PPARs and nuclear receptors, like RXRG, is the key activator of target genes involved with fatty acid oxidation, insulin secretion, and inflammation^7,13^. The relationship of PPAR-RXRG with energy imbalance underlies metabolic abnormalities, such as diabetes and chronic inflammation^34^. Moreover, FOXA3 participates on the pathway maturity onset diabetes as well as carbohydrate and glucose homeostasis. In addition, SREBF2 plays a role in energy metabolism and also lipid storage, regulation of sterol and cholesterol transport, lipid and cholesterol homeostasis. The protein coded by this gene regulates the cholesterol biosynthesis^7^. Cholesterol is the precursor of all steroid hormones and it plays a role in early fetal development by moderating nuclear receptors^35^.

Besides the imbalances in energy homeostasis, maternal nutrient restriction has been associated with an inflammatory response in the dam and the offspring^36^. We identified *TGFB1* and *HDAC8* differentially co-expressed and positively correlated in RES fetuses. The *TGFB1* gene encodes a multifunctional cytokine with immunoregulatory and anti-inflammatory properties^37^. It is also important in pregnancy maintenance^37^, hepatic stellate cell activation, and hepatic fibrosis^38^. An increased expression of MEF2D in activated hepatic stellate cells in liver and the fibrotic response was reported previously^38^. In the current study, we identified the *MEF2D* gene as a key TF, more connected in RES, and positively correlated with *TGFB1* and *HDAC8*. The TGFB1 and MEFD2 proteins have been associated with liver fibrosis activation^38,39^. Likewise, HDAC8 protein inhibition suppresses TGFB1-induced genes in pulmonary fibrosis^40^.

In response to cellular stress, HSF1 and HSF2 TFs activate genes encoding heat shock proteins (HSPs) to stabilize nascent proteins^41^. These TFs in liver showed high connectivity in RES fetuses. Since misfolded proteins are eliminated either by ubiquitination or autophagy^41^, the over-representation of the ubiquitin-mediated proteolysis pathway found here is likely acting in concert with the HSPs. Thus, these evidences suggest that RES fetuses may have shown hepatic cellular stress in response to lipid metabolism imbalances.

Maternal nutritional insults may have more dramatic effects on muscle development as it has low priority in nutrient partitioning, resulting in greater concern due to the economic importance of muscle tissue^12^. Offspring of nutrient-restricted dams showed a reduced number of muscle fibers and muscle mass, as well as altered muscle function and gene regulation^1,12,17,42^. Among the TS genes, well known upstream regulators of myogenesis, such as *MYOG, MYOD1, PAX1,* and *MYF5*^43^*,* were found by our approach. Interestingly, some of these TS regulators along with DEGs were negatively correlated with the *ZBTB33* and *ZNF131* TFs in the RES group. The *ZBTB33* gene encodes a methyl-CpG-binding protein (Kaiso) that recognizes both unmethylated and methylated CpGs, acting as a transcriptional repressor^44^. The role of Kaiso in regulating myogenic genes is still to be examined; however, Ruzov et al.^45^ showed that Kaiso is necessary during early *Xenopus laevis* development. Moreover, CIBZ, a Kaiso-like family member, represses *MYOG* in a methylation-dependent manner^44^. Although we do not have the methylation profile for these animals, Crouse et al.^30^ reported reduced methionine and increased homocysteine concentrations in amniotic fluid and serum, respectively, in RES heifers. The imbalance in one-carbon metabolism would likely affect the gene transcription by altering chromatin remodeling and DNA methylation^46^.

Although *ZNF131* acts as a transcriptional activator, Kaiso, a transcriptional regulator, was implicated in its repression, and together they fine-tune the transcription of Kaiso targets^47^. Muscle structure development and skeletal muscle tissue growth were among the BP over-represented by the 34 co-regulated genes between *ZNF131* and *ZBTB33*. The myogenic regulatory factors *MYOD1*, *MYF5*, and *MYOG* were negatively and differentially correlated with *ZBTB33* and/or *ZNF131* in RES fetuses. The *CDKN1A* gene was negatively correlated with these TFs in the RES group and also DEG^17^. The protein encoded by the *ZNF131* gene has been associated with skeletal muscle atrophy^48^. Moreover, *CDKN1A* gene expression is synergistically activated by *SMAD4*^49^ that was identified here as DK and negatively co-expressed with *MYOG* and *MYOD1*. The protein encoded by *SMAD4* has an inhibitory effect on protein synthesis and muscle growth^50^. Genes encoding proteins involved in muscle contraction, such as those from the troponin and myosin families, were also negatively regulated in the RES group. We collected the fetal tissues on day 50 of pregnancy, which coincides with the beginning of primary myogenesis^12^. Therefore, our findings show the potential mechanism by which maternal nutrient restriction negatively modulates myogenic developmental programming in cattle.

We found the DC genes from the fetal muscle over-represented in the nutrient-sensing signaling pathways PI3K-Akt, MAPK, and FoxO. These pathways along with mTOR, play a major role in muscle development, energy metabolism, gene expression, and metabolic epigenetic programming^50,51^. The Akt-mTOR pathway acts as positive regulator of protein synthesis, whereas FoxO signaling is required for muscle atrophy^50^. We found the *ELK3* TF more connected and negatively and differentially correlated with *AKT1* in fetuses from RES dams. The ELK3 protein is a transcriptional repressor whose knockdown induced the activation of the PI3k/Akt/mTOR pathway in breast cancer cell line^52^. In addition, intrauterine growth restriction in rats has been associated with reduced activity of this pathway^53^. We found *FoxO6* significantly more connected in the RES group. FoxO induction leads to the transcription of atrophy-related genes^50^. Additionally, SMAD3/SMAD4 heterodimers may repress mTOR^50^. We identified the *SMAD4* gene negatively co-expressed with *EIF4EBP1* that acts as protein synthesis inhibitor^50^. Altogether, it seems that the TFs were acting redundantly and coordinately to repress muscle formation and differentiation in response to nutrient restriction.

Like the muscle, our findings evidenced a severe impact of a moderate maternal nutrient restriction in the day 50 fetal brain genes involved with development and metabolism. First, we found a large number of DC genes between CON and RES, and several DK TFs. Second, pathways such as PI3K/AKT and mTOR seem to be differentially regulated between the CON and RES groups. The brain plays a central role in the body’s energy homeostasis by integrating metabolic signals from other tissues and coordinating adaptive changes under nutrient deficiency^54^. These signals are integrated by the mTOR pathway that together with the PI3K/Akt signaling pathway governs neuronal fate by regulating protein synthesis and energy homeostasis^55,56^. Furthermore, insulin signaling plays a role in neuronal differentiation by activating mTOR^55^. Nutrient restriction has been associated with reduced transcription rate modulated by mTOR^57^. We found the ribosome pathway over-represented by the DC genes encoding ribosomal proteins, and intriguingly these genes were mainly negatively correlated in the RES group. Impaired ribosome biogenesis has been associated with neurodevelopmental disorders and reduced protein synthesis^57,58^. The *PAK5*, *ELAVL4*, and *ZNF207* genes were DC with the ribosomal genes identified in the current study. The *ELAVL4* gene encodes an RNA-binding protein (HuD) important for neuronal development and plasticity, acting on mRNA stability, translation, and alternative polyadenylation of target genes^59^. The *PAK5* gene encodes a brain-specific protein involved with neurite development^60^. Furthermore, the PAK family members are regulators of PI3K and via the PI3K/Akt pathway modulate HuD abundance post-transcriptionally^59^. The ZNF207 TF plays a role in cell pluripotency by regulating neuronal TFs^61^.

Metabolic stress resulting from energy restriction can activate the transcription of inflammatory genes^57^. The TGF-β family is pivotal for organogenesis, and it was shown to negatively affect brain development by inhibiting neurite elongation^62^. We identified *TGFB1* and *TGFB3* DC and negatively correlated with *NKX2*-1, *ELAVL4*, and *SLC17A6* in the RES group. The *SLC17A6* gene encodes a glutamate transporter protein^63^, while *NKX2*-1 encodes a TF that regulates target genes involved with the development of interneurons^64^. In response to inflammatory processes, hypoxia-inducible factors (HIFs) modulate metabolic homeostasis by activating hypoxia-induced genes^65^. Additionally, these genes underlie cell differentiation and vascular development in the fetal brain^65^. We identified the HIF pathway over-represented from the DC genes. Among them, the *ZNF395* TF was positively correlated with most of the gene pairs DC in the RES fetuses. *ZNF395* is targeted by the HIF-1α TF activating hypoxia-induced pro-inflammatory genes^66^. On the other hand, the *ETS2* and *NFKB2* TFs that were both DC and DK showed mainly negative correlations in the RES group. The ETS2 protein has been associated with neuronal apoptosis and neurodegeneration in neurons of Down syndrome^67^. However, in macrophages, it had a negative effect on the inflammatory response^67^. Regarding the NFKB2 protein, it has a pleiotropic effect and acts in processes such as synaptic plasticity under normal physiology; however, in disease, it is involved with neuroinflammation as well as neuronal cell death^68^. Considering the findings, the moderate maternal dietary restriction induced changes in gene wiring and expression in the brain may have altered BP related to neuronal development and energy homeostasis. Although the effects of these changes in animal health and performance are still unknown, they could affect animal reactivity, stress responses, and feeding behavior^17^.

Our findings reinforce that fetal metabolic programming is affected by maternal nutritional imbalances even in early pregnancy. Energy metabolism-related pathways were over-represented in the cerebrum, liver, and muscle, suggesting a multi-tissue coordinated adaptive response mediated by differential gene expression. Likewise, pathways related to myogenesis seems to be negatively regulated in nutrient restricted fetuses. Factors affecting fetal muscle development can reduce animal performance, muscle growth, and consequently, meat production and quality^12^. Furthermore, fetal adaptive response by affecting insulin metabolism may adversely affect animal feed efficiency. It’s worth mentioning that due to the similarities underlying embryonic development in humans and cattle and the conserved genetic architecture of complex traits in both species^69^, our findings can help understanding the effects of maternal nutrition on human fetal development. Obesity, molecular disorders, and cardiovascular diseases later in life have been associated with pre-natal maternal stressors^8,70^. Pathways related to human diseases, such as maturity onset diabetes of the young and non-alcoholic fatty liver disease, were over-represented among the differentially regulated genes in our study. These findings reinforce the potential adverse effects of early maternal nutrient restriction on offspring health. Additionally, it suggests that pathways affected by maternal nutrition in cows are likely to be regulated similarly in humans.

Altogether, our data suggest that nutrient restricted animals will later in life be metabolically compromised, leading to long-term consequences probably across generations. Although the genetic basis of fetal programming is still under investigation, our results point to the key role of epigenetic mechanisms driving the genome regulation. We hypothesize that RES fetuses have impaired one-carbon metabolism, as demonstrated by the reduced availability of methionine in allantoic fluid^30^. Further research is warranted to investigate the role of non-coding RNAs and histone modifications in addition to DNA methylation as the mechanisms underlying fetal programming and the transmission of intergenerational effects.

## Conclusions

Our approach identified the complex gene network on day 50 of gestation that underlies cross-tissue communication in response to maternal nutritional restriction. We identified major regulators driving gene expression and found differences in the regulatory mechanisms involved with developmental programming between fetuses gestated in nutrient restricted and control dams. In particular, moderate dam nutrient restriction led to differential regulation of myogenic factors by the *ZBTB33* and *ZNF131* TFs that may negatively affect myogenesis. In addition to tissue-specific pathways, the over-representation of nutrient-sensing signaling pathways across tissues, such as mTOR, PI3K/Akt, and insulin, shed light on their role in fetal programming. Further research is still needed to determine the role of epigenetics and chromatin remodeling in developmental programming, their interplay with nutrition, and their impact on animal health and performance.

## Methods

### Animals, tissue collection, library preparation, and sequencing

All experiments and methods were performed in accordance with relevant guidelines and regulations. The experimental design, animal management, and tissue collection were approved by the North Dakota State University Institutional Animal Care and Use Committee and previously reported by Crouse et al.^17^. In brief, 14 Angus-cross heifers with average initial body weight = 313 ± 24.9 kg were randomly assigned at breeding to receive dietary intake of either 100% of energy requirement^71^ for 0.45 kg/day of body weight gain (control; CON, *n* = 7) or were fed a diet to delivery 60% of CON intake (restricted; RES, *n* = 7). The diet was delivered via total mixed ration (TMR), and consisted of grass hay, corn silage, alfalfa haylage, grain, and mineral mix. Dried distillers grains with solubles (53.4% NDF, 31.3% CP) were supplemented in addition to the TMR and fed to achieve the target nutrient content of the CON and RES diets^17^. The two nutritional levels supplied to the heifers were chosen to represent two nutritional states (weight gain versus maintenance) as well as conditions applicable to beef production systems.

On day 50 of gestation, cerebrum (*n* = 7/group), liver (*n* = 7/group), and muscle (*n* = 7/group) were collected from fetuses through ovariohysterectomy^72^. Tissue collection was performed at this time because fetal organogenesis have already finished and it is in the peak primary myogenesis^12^. The tissues were snap-frozen in liquid nitrogen, stored at − 80 °C, and later on, RNA isolation and sequencing were performed by the University of Minnesota Genomics Center (Minneapolis-St. Paul, MN), as reported elsewhere^17^. After RNA quality control, strand-specific RNA libraries were prepared, and sequencing was carried out on the Illumina HiSeq 2500 platform. Sequencing was pair-end (50-bp reads) at a depth of 2 × 10.4 M reads/sample in both forward and reverse directions^17^. Fetal sex was determined by PCR based on the hind limb DNA amplification of the *DDX3X* and *DDX3Y* genes located on the X and Y chromosomes. The PCR product was analyzed on agarose gel electrophoresis, where males showed two bands, and females resulted in only one. This analysis was based on the primers, and PCR parameters reported elsewhere^73^. Thus, were evaluated 6 males and 1 female in the CON group and 5 males and 2 females in the RES group.

### RNA-Seq data analyses and differentially expressed genes

Data quality control was performed with FastQC v.0.11.8^74^ (https://rb.gy/lxqcwa) and MultiQC v.1.8^75^ (https://multiqc.info/) software. Filtered reads were mapped to the bovine reference genome (ARS-UCD 1.2)^76^ and gene annotation file (release 100) from the *Ensembl* database. The STAR aligner version 2.7.3a^77^ (https://rb.gy/dlgdva) using the *–quantMode* GeneCounts flag was used to obtain the raw counts per gene.

The RStudio^78^ v. 1.1.442 environment for R^79^ v.3.5.1. was used for data analyses as described below. Post-mapping quality control was performed using MultiQC, NOISeq v.2.26.0^80^ (10.18129/B9.bioc.NOISeq), and edgeR v.3.24.0^81^ (10.18129/B9.bioc.edgeR). Read counts were transformed to counts per million (CPM), and lowly expressed genes (CPM < 1 in 80% of the samples) were filtered out. Gene expression normalization was performed under two approaches: (1) across all samples and the three tissues together and (2) across all samples but individually for each tissue. Both procedures were performed using the *VST* function from DESeq2 v.1.22.1^82^ (10.18129/B9.bioc.DESeq2). To control gene expression for sex effect, fetal sex was included in the DESeq2 model as a factor. Hierarchical clustering and Principal Component Analysis on normalized data were performed by using NOISeq v.2.26.0^80^ and DESeq2 v.1.22.1^82^. Normalized gene expression (*n* = 17,164) from the first approach was used to predict TS genes, to predict the key regulators (TFs), and to build the tissue-to-tissue network. On the second approach, besides removing genes with low expression based on the CPM criterion, genes with low dispersion after normalization were filtered out when the quantile dispersion measure was < 0.2. Further, the normalized data were used for tissue condition-specific networks construction, differential connectivity, and differential gene co-expression analyses. A summary of the workflow is shown in Fig. 1.

Differential expression analysis for the tissues and animals used here were reported elsewhere^17^. In brief, by using the Tuxedo Suite pipeline, the authors identified 373 DEGs between the CON and RES groups for fetal liver (*n* = 201), muscle (*n* = 144), and cerebrum (*n* = 28). Herein, these DEGs were used for further analyses.

### Identification of tissue-specific genes (TS)

The normalized expression was used to calculate a mean value for each gene from each tissue separately. To summarize the gene specificity among tissues, the Tau index (τ) was computed using the R-package tispec v.0.99.0^83^ (https://rb.gy/ch0h4w) considering the following Eq. ^23^:

$$\tau = \frac{{\mathop \sum \nolimits_{i = 1}^{n} \left( {1 - \hat{x}_{i} } \right) }}{n - 1 }$$

in which *n* is the number of tissues and $x_{i}$ is the gene expression in tissue *i*. Tau values range from zero (genes ubiquitously expressed) to one (TS genes)^84^. Accordingly, we defined the TS genes with τ ≥ 0.8^84^.

To support the gene tissue specificity analysis, we performed a TS over-representation analysis using TopAnat (https://rb.gy/6qvwla). This tool allowed us to identify where genes from a set are preferentially expressed^85^, considering the anatomical structures and the Uberon ontology annotations^86^. Thus, for each TS gene list, we used TopAnat based on bovine as the target species and the Begee database for *B. taurus* as background^85^.

### Identification of key transcription factors (TFs)

To identify TFs differentially regulating gene expression in CON and RES groups for each tissue, we used the regulatory impact factor (RIF) algorithms, RIF1 and RIF2^24^. These metrics assume that master regulators in a network change their behavior in different biological conditions contributing to differential gene expression^19,26^. RIF algorithm was implemented in FORTRAN 90 based on the source code available from the RIF’s main author^24^. The RIF1 score highlights regulators differentially connected to the targets, whereas RIF2 measures those TFs with the potential to act as predictors of target abundance^24,26^. To identify the regulators, we first downloaded 1396 TFs from the Animal Transcription Factor Database (Animal TFDB v3.0)^87^. After filtering out those TFs not expressed in our dataset, 1164 TFs were contrasted to a unique list of DEG and TS genes. The RIF comparisons were performed separately for each tissue, comparing CON *versus* RES groups.

### Gene networks

To determine the co-expression profile of gene pairs across tissues and experimental groups, we used the partial correlation and information theory (PCIT) algorithm^28^. FORTRAN 90 source code to perform the PCIT algorithm is available as Supplementary File 1 at https://rb.gy/vyfqrv. This approach compares all possible triplets of genes by exploring the concepts of partial correlation and mutual information reporting the significantly correlated pairs after accounting for all the other genes in the network^19,28,88^. For the tissue-to-tissue network inference, all the genes (*n* = 1,160) selected as DEG, TS genes, or TFs from the previous analyses were used considering all animals and tissues together. Significant co-expressed pairs were determined based on partial correlation values greater than |0.9| (*p* < 0.01).

To shed light on the coordinated gene expression and identify the conditional regulatory relationships underlying the differences between RES and CON groups, a differential gene co-expression network analysis was performed by using the R-package DGCA v.1.0.2^22^ (https://rb.gy/qadgby). To this end, all the genes kept after filtering and normalization (cerebrum, *n* = 11,432; liver, *n* = 10,146; and muscle, *n* = 11,439) were used. Significantly correlated gene pairs were determined based on the adjusted p-values (*q* ≤ 0.05). The DC gene pairs were grouped in one of the eight differential correlation classes proposed by McKenzie et al.^22^. These classes (+/+ ; +/− ; +/0; − /− ; −/+ ; −/0; 0/+ ; 0/−) show the correlation signal [positive, negative, or not significant (0)] for each gene and condition when contrasting the groups (CON *versus* RES).

To identify changes in the gene network topology between conditions, we used PCIT to build CON and RES networks separately for each tissue from the same dataset used for DGCA analysis. Significantly correlated pairs were selected when DEGs or TFs were present and showed a partial correlation greater than |0.9| (*p* < 0.01). To explore the DK genes between the maternal dietary intake conditions, we first standardized the connectivity (K) measures for each network by dividing each gene connectivity by the maximum connectivity^25^. Next, the DK measure was defined as $DK_{i} = K_{CON} \left( i \right) - K_{RES} \left( i \right)$. To assign a significance level, each score was transformed into a *Z-score,* and values located ± 1.96 from the standard deviation (SD) were considered significant (*p* < 0.05). The DyNet^27^ Cytoscape plugin v.1.0 (https://rb.gy/9vp9js) was used to visualize the most rewiring nodes between the conditions considering the Dn-score. This score ranks the variability of connections and highlights the most rewired nodes based on a central reference network^27^ constructed from the union of RES and CON networks. Networks were visualized in Cytoscape (v.3.7)^89^ (https://cytoscape.org/). Highly connected genes or “hubs” were defined considering the degree measure (2 SD, *p* < 0.05) retrieved from the Cytoscape Network Analyzer tool v.2.7^90^ (https://rb.gy/twossl).

### Functional over-representation analyses

We carried out functional over-representation analyses to identify the biological process and pathways underpinning relevant functions related to fetal programming. The gene lists from DEG, TS, and TF analyses were queried by using ClueGO v. 2.5.7 (https://rb.gy/x5cb7q), considering a cluster analysis framework^91^. This approach allowed us to underline specific and common biological functions within and among gene lists for each tissue. To reduce the redundancy of the GO terms, we applied the fusion of similar biological processes. Likewise, the terms were grouped based on the kappa score = 0.4. Significant results were taken considering the group *p*-value after Bonferroni step down correction (*adj.pvalue* ≤ 0.05). Pathway analysis was performed for DC genes by using the ShinyGO v0.61^92^ (https://rb.gy/o97axc). This analysis was performed separately for each tissue considering the *B. taurus* annotation as background and *adj.pvalue* ≤ 0.05 as significant.

## Supplementary Information

Supplementary Figures.

Supplementary Table.

Supplementary Table.

Supplementary Table.

Supplementary Table.

## Data Availability

All relevant data are within the paper and its Supplementary Information files. All sequencing data is publicly available on NCBI’s Gene Expression Omnibus through GEO Series accession number GSE154299 (Bioproject PRJNA645822). All additional datasets generated and analyzed during this study are available from the corresponding author.
