# Supplementary material for: Cerebrum, liver, and muscle regulatory networks uncover maternal nutrition effects in developmental programming of beef cattle during early pregnancy
Source: Sci Rep. 2021 Feb 2;11:2771. doi: 10.1038/s41598-021-82156-w (PMC7854659; doi:10.1038/s41598-021-82156-w)
Supplement: Supplementary file 1 — Supplementary Figures. [file 41598_2021_82156_MOESM1_ESM.pdf]

Cerebrum, liver, and muscle regulatory networks uncover maternal nutrition effects in developmental programming of beef cattle during early pregnancy

Wellison J. S. Diniz[1*], Matthew S. Crouse[2], Robert A. Cushman[2], Kyle J. McLean[3], Joel S. Caton[1], Carl R. Dahlen[1], Lawrence P. Reynolds[1], Alison K. Ward[1]

[1]Department of Animal Sciences, North Dakota State University, Fargo.
[2]USDA, ARS, U.S. Meat Animal Research Center, Clay Center, NE.
[3]Department of Animal Science, University of Tennessee, Knoxville, TN
*Corresponding author: w.dasilvadiniz@ndsu.edu

SUPPLEMENTARY FIGURES

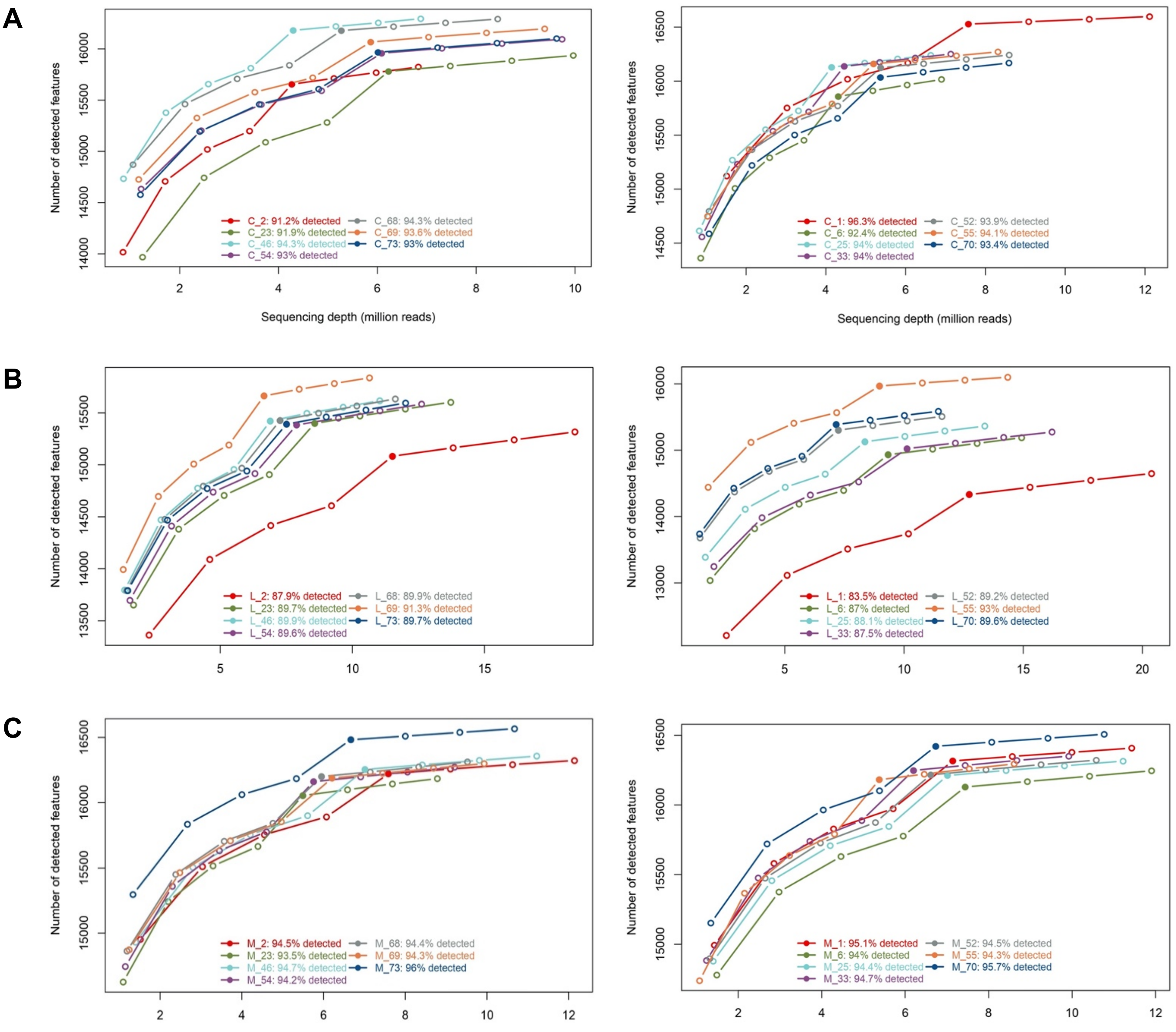

Figure S1. Saturation curve of the cerebrum (A), liver (B), and muscle (C) RNA-Seq data. The plot shows the number of detected genes represented as full dots (y-axis) and the new detections per each million of additional reads sequenced (x- axis) at increasing sequencing depths for different samples after removing the lowly expressed genes. The plot was created using NOISeq v.2.26.0 on R environment.

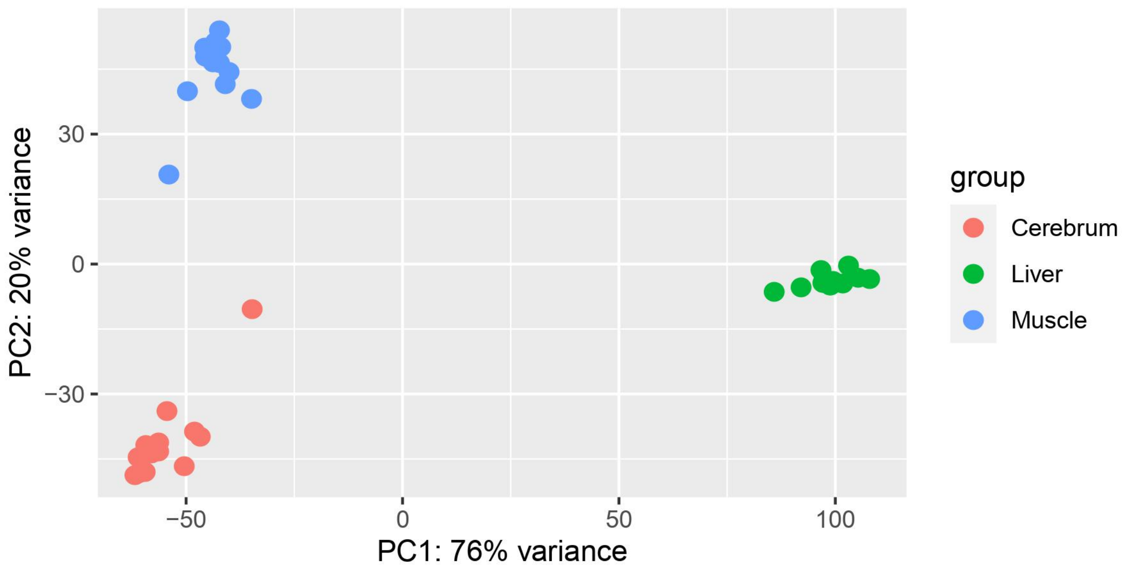

Figure S2. PCA analysis from RNA-Seq data of 17,164 genes across three tissues and 42 samples. Each dot represents one tissue from one animal. The plot was created using DESeq2 v.1.22.1 on R environment.

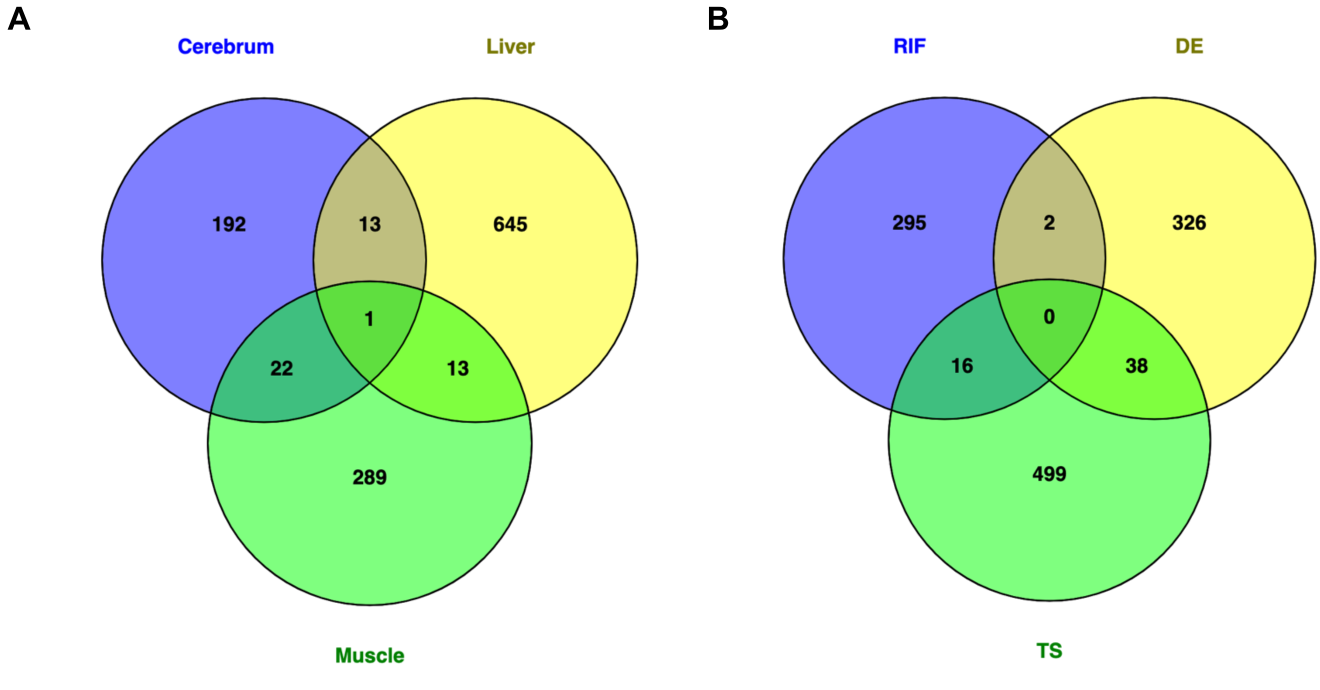

Figure S3. Venn diagram of 1,160 unique genes used for tissue-to-tissue network construction overlapped by tissue **(A)** and category **(B)**. The genes from cerebrum (*n* = 228), liver (*n* = 672), and muscle (*n* = 325) were prioritized considering the following criteria: (1) differentially expressed between RES and CON groups; (2) tissue-specific; and (3) key TFs based on RIF1 or RIF2. Venn diagram was created using Venny v.2.1. (https://rb.gy/jxxufy).

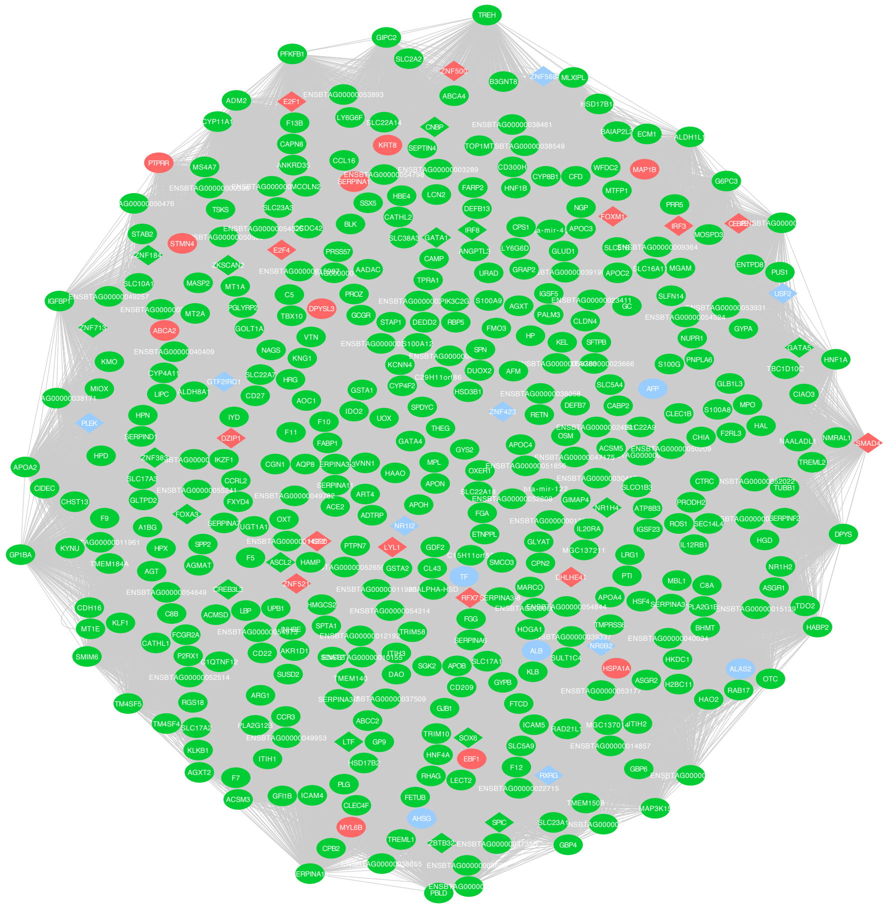

Figure S4. Gene co-expression sub-network with 427 nodes and 81,308 interactions from the first neighbors of the *GATA1*, *HSF2*, and *PLEK2* transcription factors. Only nodes with a correlation greater than |0.9| are shown. Nodes are colored considering their tissue (cerebrum, liver, and muscle). Overlapped genes between analysis were colored based on the tissue with its maximum expression. Transcription factors are represented by a diamond shape. Gene network was created on Cytoscape v.3.7.

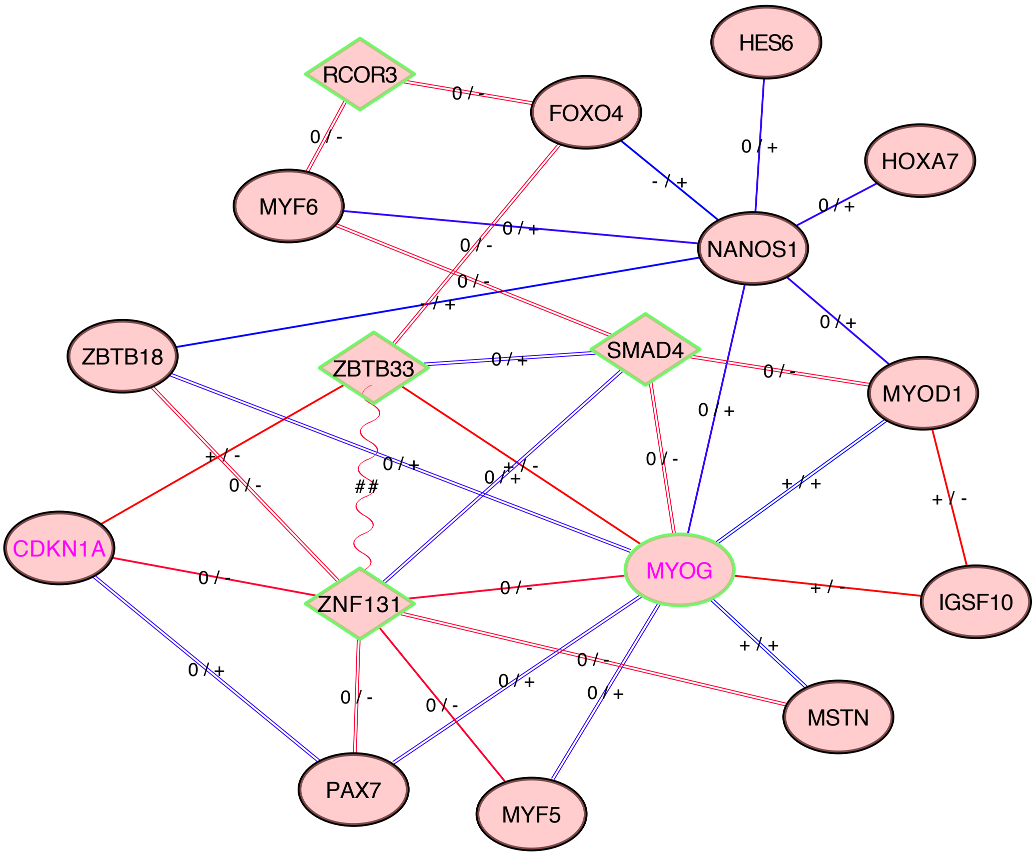

Figure S5. Gene co-expression network of myogenic factors. Differentially expressed nodes are labeled in pink. Differentially connected nodes have green borders. Transcription factors are represented by a diamond shape. Single line edges are based on the differential gene co-expression analysis, whereas the double lines are based on the tissue condition-specific network (PCIT). Red and blue lines represent negative or positive correlation between CON and RES groups, respectively. ##based on Robinson et al. (2017, Doi: 10.1016/j.bbrc.2017.09.007). Gene network was created on Cytoscape v.3.7.

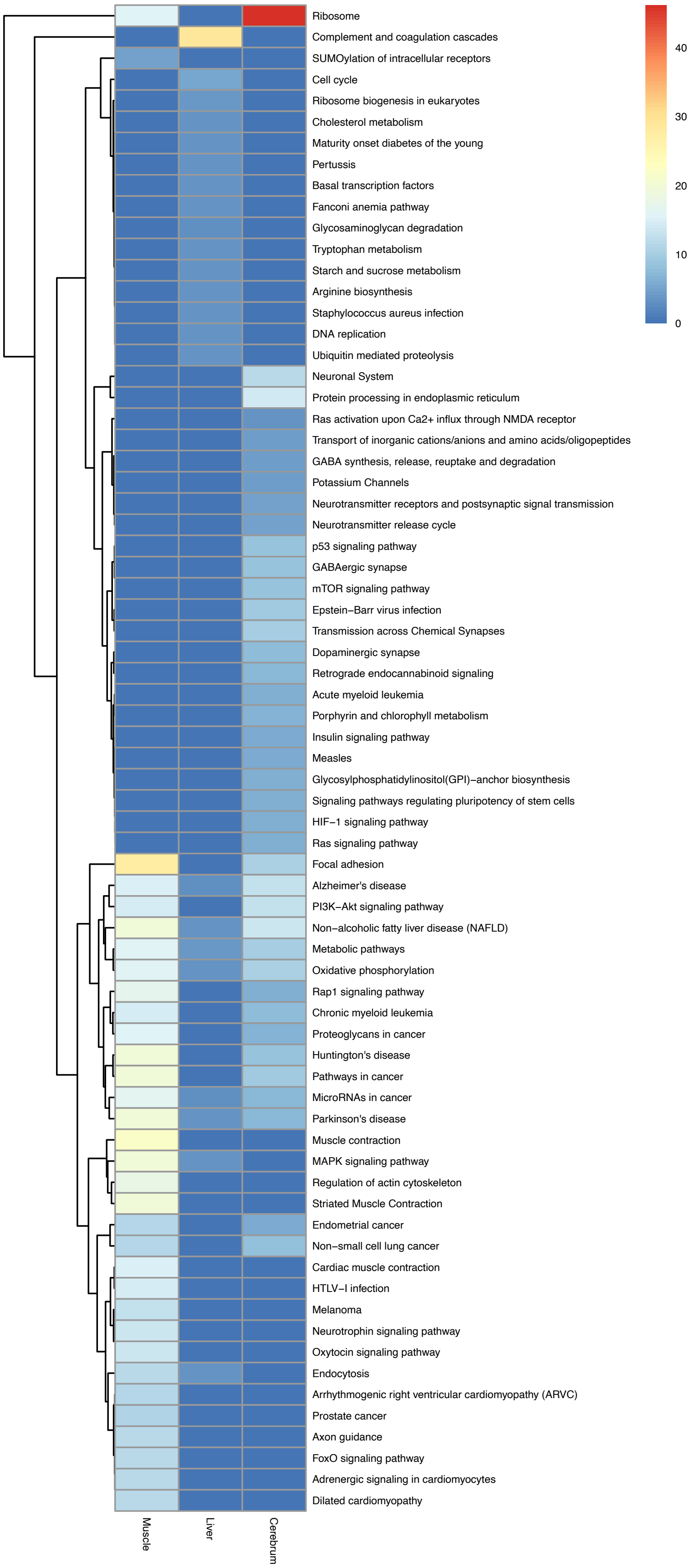

Figure S6. Heat map of over-represented signaling pathways of co-expressed genes from the cerebrum, liver, and muscle tissues. The matrix is color-coded based on the –log10 (adjusted p-value) according to the legend. Heatmap was constructed using pheatmap v.1.010. on R.
